# Position and Attitude Control of Multi-Modal Underwater Robots Using an Improved LADRC Based on Sliding Mode Control

**DOI:** 10.3390/s25196010

**Published:** 2025-09-30

**Authors:** Luze Wang, Yu Lu, Lei Zhang, Bowei Cui, Fengluo Chen, Bingchen Liang, Liwei Yu, Shimin Yu

**Affiliations:** 1 The College of Engineering, Ocean University of China, Qingdao 266100, China; wangluze001119@163.com (L.W.); cbw@stu.ouc.edu.cn (B.C.); bingchen@ouc.edu.cn (B.L.); yuliwei@ouc.edu.cn (L.Y.); yushimin@ouc.edu.cn (S.Y.); 2 China Merchants Marine and Offshore Research Institute Co., Ltd., Shenzhen 518067, China; luyu7@cmhk.com (Y.L.); chenfengluo@cmhk.com (F.C.)

**Keywords:** multi-modal underwater robots, depth control, heading control, LADRC, sliding mode control, SM-ADRC

## Abstract

This paper focuses on the control problems of a multi-modal underwater robot, which is designed mainly for the task of detecting the working environment in deep-sea mining. To tackle model uncertainty and external disturbances, an improved linear active disturbance rejection control scheme based on sliding mode control is proposed (SM-ADRC). Firstly, to reduce overshoot, a piecewise fhan function is introduced into the tracking differentiator (TD). This design retains the system’s fast nonlinear tracking characteristics outside the boundary layer while leveraging linear damping within it to achieve effective overshoot suppression. Secondly, two key enhancements are made to the SMC: an integral sliding surface is designed to improve steady-state accuracy, and a saturation function replaces the sign function to suppress high-frequency chattering. Furthermore, the SMC integrates the total disturbance estimate from the linear extended state observer (LESO) for feedforward compensation. Finally, the simulation experiment verification is completed. The simulation results show that the SM-ADRC scheme significantly improves the dynamic response and disturbance suppression ability of the system and simultaneously suppresses the chattering problem of SMC.

## 1. Introduction

The development and utilization of the ocean have become a key focus of research and attention in fields such as scientific research and environmental monitoring [1,2]. Remotely operated vehicles (ROVs) and autonomous underwater vehicles (AUVs) are representative types of underwater robots. As essential tools for ocean development and exploration, they play a crucial role in applications like maritime rescue, salvage, nearshore exploration, and seabed mining [3,4,5].

With the development of the underwater robot field, research on multi-modal underwater robots has received extensive attention. Multi-modal characteristics endow them with multi-environmental adaptability and multi-functionality, which broadens their potential application scenarios [6,7,8]. At present, multi-modal underwater robots integrate various movement methods, such as wheeled, legged, and thruster drives, and are capable of moving flexibly in complex underwater environments [9,10]. X. Ma et al. [11] developed a lightweight micro-robot that adopts a propeller-leg composite structure. By adjusting the folding angle of the body to change the thrust direction of the propeller, the single-actuator robot is endowed with underwater movement capability. However, its strong coupling and nonlinear dynamic characteristics make precise control of the propulsion direction extremely challenging. Shen et al. [12] integrated multiple mechanisms such as wheels, legs, and paddles and designed a new type of eccentric paddle composite structure. This design endows the robot with multiple motion modes and expands its application range. However, this inevitably brings about the control challenges of multi-modal smooth switching and the complex coordination of actuators. The enhanced flexibility and environmental adaptability brought about by multi-modal characteristics also pose higher requirements for the design of its control system.

The motion control technology of underwater robots is the core guarantee for achieving complex operations such as stable hovering and precise trajectory tracking [13,14,15]. In tasks such as marine mining inspection, robots need to maintain the stability of high-precision poses in strongly disturbed environments, such as fixed depth and heading. However, their motion exhibits inherently nonlinear and strongly coupled characteristics, further complicated by challenges like ocean current disturbances and model uncertainties. These factors cause traditional control methods to suffer from low steady-state accuracy, lagging dynamic response, and an insufficient ability to suppress sudden disturbances [16,17]. These problems constitute the key bottlenecks for achieving high-quality control of underwater robots.

As a classic control method, the PID control method has a simple structure and is insensitive to external disturbances and model parameter errors [18,19,20]. It is widely used in the control of various underwater robots. However, due to the nonlinear strong coupling characteristics of multi-modal underwater robots and the presence of complex disturbances, PID control is difficult to guarantee good quality. Active disturbance rejection control (ADRC) actively compensates for disturbances through an extended state observer [21,22], providing a new approach for enhancing the control accuracy and disturbance rejection capability of underwater robots. Wang et al. [23] designed a control scheme for an improved LADRC based on the Nutcracker optimization algorithm (NOA) for the automatic depth control problem of ROVs in complex disturbance environments. This enables the automatic setting of LADRC parameters and rapid, high-precision depth control. Zhang et al. [24] proposed an improved active disturbance rejection control method for the depth tracking problem of autonomous underwater robots. This method introduces the Nefal nonlinear function optimization state observer and feedback controller. Compared with the traditional active disturbance rejection control method, it has significantly improved tracking performance and anti-disturbance ability. Arcos and Gutierrez [25] proposed a predictive control method based on an active perturbation robust model for the three-dimensional tracking problem of cabled underactuated underwater robots. This scheme combines active disturbance rejection control with robust control. Compared with traditional robust control methods, both trajectory tracking and anti-disturbance performance have been improved to a certain extent. Although ADRC can significantly enhance the anti-disturbance ability of underwater robots, it still has some limitations. The noise amplification effect of its tracking differentiator (TD) is prone to cause steady-state error, and the observation lag of its extended state observer (ESO) to sudden disturbances limits the dynamic response performance.

Sliding mode control (SMC) has been introduced as a nonlinear control method with strong robustness. It forces the system state trajectory onto a predefined sliding surface and maintains it there. This mechanism enables stable control despite parameter uncertainties and external disturbances, while also providing the ability to rapidly suppress sudden disturbances. Qiao and Zhang [26] proposed a second-order fast non-singular terminal sliding mode controller for the three-dimensional trajectory tracking problem of ODIN underwater robots. Compared with the non-singular terminal sliding mode control method, this method has faster convergence and robustness. However, the prominent drawback of sliding mode control is that the moving points fluctuate within a certain range on the sliding mode surface, which may lead to chattering in the system [27]. Soylu et al. [28] proposed a chattering sliding mode control method, mainly by designing an adaptive control term to replace the discontinuous switching control term. Adaptive control terms can continuously compensate for the uncertain influence of the system model, thereby eliminating chattering. However, this method is complex and involves many control parameters, making it difficult to implement in practice. Ali et al. [29] proposed a scheme combining a finite-time extended observer with non-singular terminal sliding mode control in response to various hydrodynamic uncertainties and external environmental disturbances. This scheme reduces the chattering of the sliding mode surface and improves the response speed and anti-disturbance performance of the underwater robot system. Xu et al. [30] proposed a scheme combining the robust prescribed-time extended state observer (RPTESO) and the non-singular robust practical predefined-time sliding mode control (RPPSMC) for the trajectory tracking problem under the uncertainty and disturbance of AUV models. This scheme designs an adaptive law on the premise of ensuring convergence speed, significantly improving trajectory tracking accuracy in complex environments.

This paper focuses on the control problems of a multi-modal underwater robot under model uncertainty and external disturbances, and proposes an improved linear active disturbance rejection control scheme based on sliding mode control (SM-ADRC). By integrating the disturbance estimation capability of the linear extended state observer (LESO) with the strong robustness of sliding mode control, this scheme innovatively designs an anti-disturbance and anti-saturation architecture. This design enhances the dynamic response and disturbance suppression ability of the system and can also suppress chattering. The effectiveness of the scheme is ultimately confirmed through simulation.

The main contributions of this article include the following:

1. Based on the ADRC theory, an SM-ADRC scheme is designed to solve the control problem of multi-modal underwater robots under model uncertainty and external disturbances.
2. A piecewise fhan function is designed in the TD. The fast nonlinear tracking characteristics of the outer layer of the boundary are retained, while linear damping is introduced within the boundary layer to suppress overshoot, effectively reducing response overshoot. In SMC, an integral sliding mode surface is designed, and the saturation function is adopted instead of the sign function. This effectively weakens the chattering phenomenon in the SMC, while maintaining the strong robustness of the system.
3. The stability of the system is proved by using Lyapunov theory. Numerical simulation experiments show that this scheme improves the robustness, dynamic performance, and steady-state accuracy of the system in vector propulsion and quadrotor modes.

The subsequent sections of this article are structured as follows: Section 2 presents the mathematical model of the multi-modal underwater robot, and Section 3 introduces the SM-ADRC control scheme, including the design of LESO, TD, and SMC. Section 4 introduces the simulation experiments in MATLAB R2023b. Finally, Section 5 summarizes the experimental results and lists potential research directions.

## 2. Modal Analysis and Mathematical Model

### 2.1. Multi-Modal Analysis

**Figure 1:**
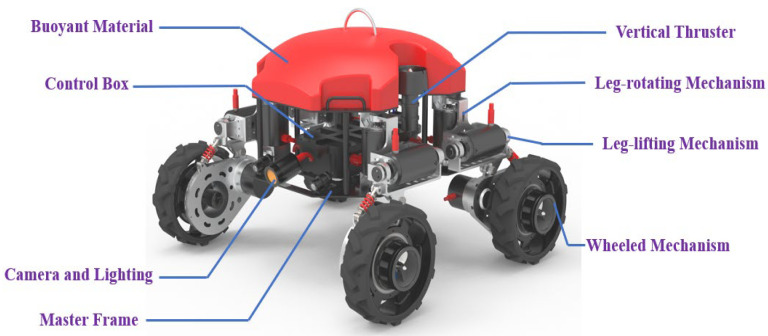
Overall structure of the multi-modal underwater robot.

The multi-modal underwater robot is specially designed for the task of detecting the working environment in deep-sea mining. It measures 1.14 m in length, 0.792 m in width, and 1.145 m in height, with a total mass of 133.5 kg. The overall structure consists of buoyant material, control box, camera and lighting, master frame, thruster, leg-rotating mechanism, leg-lifting mechanism, and wheeled mechanism, as shown in Figure 1.

**Figure 2:**
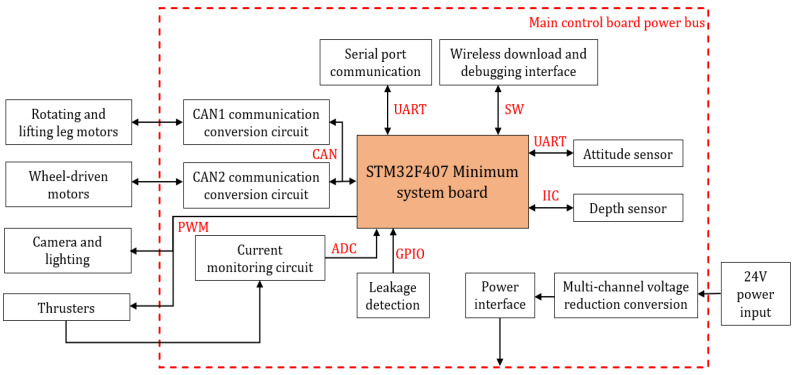
System hardware block diagram.

**Figure 3:**
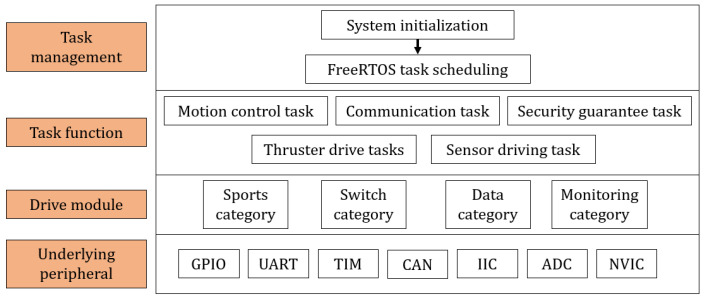
System software block diagram.

The block diagram of the hardware system is shown in Figure 2. The main control hardware system uses the MCU microcontroller as its core. It is equipped with multiple communication interfaces to interact with external devices and realizes signal processing and control through multiple circuit modules. The block diagram of the system software is shown in Figure 3. The design of the system software is based on the hardware circuit configuration and functional features of the robot. Adopting a modular and hierarchical structure, it aims to enhance the system’s scalability, reliability, and response speed. The entire software system is divided into four levels: task management, task function, driver module, and underlying peripheral. Each level has a clear functional division and closely cooperates with other levels to ensure the normal operation of the main control software system.

**Figure 4:**
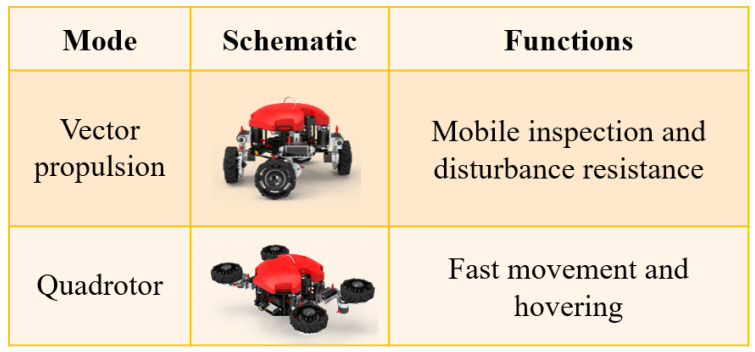
Multi-modal classification of the robot.

The multi-degree-of-freedom wheel-leg structure is the core unit for robots to achieve multi-modal switching and movement. It is composed of a leg-rotating mechanism, a leg-lifting mechanism, and a wheeled mechanism. Each wheeled mechanism is equipped with a thruster to ensure multi-directional movement after mode switching. To better describe the motion characteristics and applicable scenarios of robots, the motion modes of robots are classified based on different combinations of the leg-rotating mechanism and the leg- lifting mechanism, as shown in Figure 4. By adjusting the leg-rotating mechanism forward and backward by 45° while keeping the leg-lifting mechanism unchanged, the original propulsion system layout can be transformed into an omnidirectional vector thrust propulsion system layout. This mode is called the vector propulsion mode. Adjusting the angle of the leg-lifting mechanism to 90° and keeping the leg-rotating mechanism at 0° can change the thruster in the wheeled mechanism to a vertical direction. This mode is very similar to that of a quadrotor unmanned aerial vehicle and thus is called the quadrotor mode.

### 2.2. Kinematic Model

**Figure 5:**
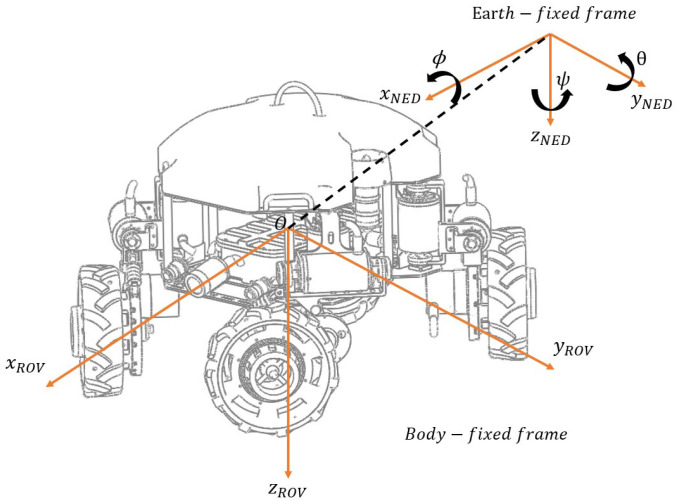
Multi-modal underwater robot coordinate system.

When multi-modal underwater robots adjust their attitudes in water, the force conditions are rather complex. It is necessary to model their motion laws to describe the changes in their positions and angles. The mathematical model in this paper is mainly modeled based on Fossen’s ocean vehicle model [31]. To standardize the description of the six-degree-of-freedom motion model, the motion parameters of the six degrees of freedom are defined. The model definition refers to the SNAME standard [32], and a standardized volumetric coordinate system and geodetic coordinate system are established. The specific coordinate representation is shown in Figure 5.

Among them, the geodetic coordinate system (*E* − *xyz*) is a fixed coordinate system, and the robot body coordinate system (*O* − *xyz*) is a moving coordinate system, which changes along with the motion state of the robot. To describe the representation of the six-degree-of-freedom motion of the multi-modal underwater robot in different coordinate systems, the expression of its motion is defined. The specific variable representations are shown in Table 1. The motion vector used to represent the position and angle of the underwater multi-modal robot in the geodetic coordinate system is η, the vector representing the linear velocity and angular velocity in the robot coordinate system is v, and the vector representing the force and moment is τ. The specific representation is shown in Equation (1).

(1) $$\eta ={\left[x\;y\;z\;\phi \;\theta \;\psi \right]}^{T}\;\;\;\nu ={\left[u\;v\;w\;p\;q\;r\right]}^{T}\;\;\;\tau ={\left[X\;Y\;Z\;K\;M\;N\right]}^{T}$$

To link the motion amounts between different coordinate systems, Euler angles are adopted for coordinate transformation. The coordinate transformation is carried out using the coordinate transformation matrix J(Φ) to establish a connection between the velocity of the motion coordinate system and the position and angle of the geodetic coordinate system. The specific representation is shown in Equations (2) and (3).

(2) $$\Phi ={\left[\phi \;\theta \;\psi \right]}^{T}$$

(3) $$\dot{\eta }=J\left(\Phi \right)\nu$$

The transformation matrix J(Φ) from the organism coordinate system to the world coordinate system is shown in Equation (4). The specific forms of $J_{1}\left(\Phi \right)$ and $J_{2}\left(\Phi \right)$ are shown in Equations (5) and (6), respectively.

(4) $$J\left(\Phi \right)=\left[\begin{array}{cc} J_{1}\left(\Phi \right) & 0_{3\times 3}\\ 0_{3\times 3} & J_{2}\left(\Phi \right) \end{array}\right]$$

(5) $$J_{1}\left(\Phi \right)=\left[\begin{array}{ccc} c_{\psi }c_{\theta } & c_{\psi }s_{\theta }s_{\phi }-s_{\psi }c_{\phi } & c_{\psi }s_{\theta }c_{\phi }+s_{\psi }s_{\phi }\\ s_{\psi }c_{\theta } & s_{\psi }s_{\theta }s_{\phi }+c_{\psi }c_{\phi } & s_{\psi }s_{\theta }c_{\phi }-c_{\psi }s_{\phi }\\ -s_{\theta } & c_{\theta }s_{\phi } & c_{\theta }c_{\phi } \end{array}\right]$$

(6) $$J_{2}\left(\Phi \right)=\left[\begin{array}{ccc} 1 & s_{\psi }t_{\theta } & c_{\phi }t_{\theta }\\ 0 & c_{\phi } & s_{\phi }\\ 0 & {s_{\phi }/c}_{\theta } & c_{\phi }{/c}_{\theta } \end{array}\right]$$

In the formula, $c_{\varphi }$, $s_{\varphi }$ and $t_{\theta }$ are the abbreviated forms of cos(φ), sin(φ), and tan(θ). Similarly, the definitions of the other trigonometric functions can be known. According to the above formula, when the pitch angle θ is ±90°, the angular velocity transformation fails and the problem of singular points occurs. Therefore, the actual pitch angle needs to meet the condition of −90°<θ<90°.

### 2.3. Dynamical Model

The dynamic equations in the motion coordinate system of the multi-modal underwater robot can be expressed in the form of Equation (7).

(7) $$M\dot{\nu }+C(\nu )\nu +D\left(\nu \right)\nu +g\left(\eta \right)=\tau$$

Here, $M\in R^{6\times 6}$ is the inertia matrix, and $M=M_{RB}+M_{A}$. $M_{RB}$ represents the rigid body mass matrix, denoted as $M_{RB}=diag(m,m,m,I_{xx},I_{yy},I_{zz})$, and $M_{A}$ is the additional mass matrix, denoted as $M_{A}=diag(-X_{\dot{u}},-Y_{\dot{v}},{-Z}_{\dot{w}},-K_{\dot{p}},-M_{\dot{q}},-N_{\dot{r}})$. Here m is the mass of the robot, $I_{xx},{\;I}_{yy},$ and $I_{zz}$ are the inertia tensors, and $X_{\dot{u}},Y_{\dot{v}},Z_{\dot{w}},K_{\dot{p}},M_{\dot{q}},N_{\dot{r}}$ are measurable hydrodynamic constants.

The Coriolis matrix C(ν) is composed of the ontological Coriolis matrix $C_{RB}$ and the additional mass Coriolis matrix $C_{A}$, that is, $C\left(\nu \right)=C_{RB}\left(\nu \right)+C_{A}\left(\nu \right)$. These two parts reflect, respectively, the inertial coupling effect of the multi-modal underwater robot body and the inertial coupling effect caused by water bodies, as shown in Equations (8) and (9).

(8) $$C_{RB}\left(\nu \right)=\left[\begin{array}{cc} \begin{array}{ccc} \;0 & \;0\; & \;0\;\\ \;0 & \;0\; & \;0\;\\ \;0 & \;0\; & \;0\; \end{array} & \begin{array}{ccc} \;0\; & mw\; & -mv\;\\ -mw\; & 0\; & mu\\ mv\; & -mu\; & \;0\; \end{array}\\ \begin{array}{ccc} 0 & mw & -mv\\ -mw & 0 & mu\\ mv & -mu & \;0 \end{array} & \begin{array}{ccc} 0 & I_{zz}r & -I_{yy}q\\ -I_{zz}r & 0 & I_{xx}p\\ I_{yy}q & {-I}_{xx}p & \;0 \end{array} \end{array}\right]$$

(9) $$C_{A}\left(\nu \right)=\left[\begin{array}{cc} \begin{array}{ccc} \;0\; & \;0\; & \;0\;\\ \;0\; & \;0\; & \;0\;\\ \;0\; & \;0\; & \;0\; \end{array} & \begin{array}{ccc} 0 & {-Z}_{\dot{w}}w & -Y_{\dot{v}}v\\ Z_{\dot{w}}w & 0 & -X_{\dot{u}}u\\ -Y_{\dot{v}}v & X_{\dot{u}}u & 0 \end{array}\\ \begin{array}{ccc} \;0 & {-Z}_{\dot{w}}w & -Y_{\dot{v}}v\\ Z_{\dot{w}}w & \;0 & -X_{\dot{u}}u\\ -Y_{\dot{v}}v & X_{\dot{u}}u & \;0 \end{array} & \begin{array}{ccc} 0 & -N_{\dot{r}}r & M_{\dot{q}}\\ N_{\dot{r}}r & 0 & -K_{\dot{p}}p\\ -M_{\dot{q}}q & K_{\dot{p}}p & 0 \end{array} \end{array}\right]$$

Hydrodynamic damping includes wave-making resistance, frictional resistance, viscous pressure resistance, and other components. For robots moving at low speeds, when considering hydrodynamic damping, a more accurate water damping model can be obtained by ignoring the damping coupling term and the damping term above the second order. The damping matrix can be expressed as Equation (10).

(10) $$D\left(\nu \right)=D_{L}\left(\nu \right)+D_{N}\left(\nu \right)$$

The linear damping matrix $D_{L}\left(\nu \right)$ and the nonlinear damping matrix $D_{N}\left(\nu \right)$ are expressed as Equations (11) and (12), respectively.

(11) $$D_{L}\left(\nu \right)=diag(X_{u},Y_{v},Z_{w},K_{p},M_{q},N_{r})$$

(12) $$D_{N}\left(\nu \right)=diag(X_{u\left|u\right|}\left|u\right|,Y_{v\left|v\right|}\left|v\right|,Z_{w\left|w\right|}\left|w\right|,K_{p\left|p\right|}\left|p\right|,M_{q\left|q\right|}\left|q\right|,N_{r\left|r\right|}\left|r\right|)$$

The center of gravity position in the body coordinate system is defined as $\left[x_{W},y_{W},z_{W}\right]$, and the floating center position as $\left[x_{B},y_{B},z_{B}\right]$. Then, the restoring force matrix g(η) is expressed as shown in Equation (13).

(13) $$g\left(\eta \right)=\left[\begin{array}{c} \begin{array}{c} \left(B-W\right)s_{\theta }\\ \left(W-B\right)c_{\theta }s_{\phi }\\ \left(W-B\right)c_{\theta }c_{\phi } \end{array}\\ \begin{array}{c} \left(y_{W}W-y_{B}B\right)c_{\theta }c_{\phi }-\left(z_{W}W-z_{B}B\right)c_{\theta }s_{\phi }\\ \left(z_{B}B-z_{W}W\right)s_{\theta }-\left(x_{W}W-x_{W}B\right)c_{\theta }c_{\phi }\\ \left(x_{W}W-x_{B}B\right)c_{\theta }s_{\phi }-\left(y_{W}W-y_{B}B\right)s_{\theta } \end{array} \end{array}\right]$$

The origin of the body coordinate system is the center of gravity of the multimodal underwater robot, and the weight-floating centers are on the same vertical line after balancing. Its center of gravity position can be written as [0,0,0], the floating center position is $\left[0,0,z_{B}\right]$, and the restoring force matrix can be further simplified to Equation (14).

(14) $$g\left(\eta \right)=\left[\begin{array}{c} \begin{array}{c} \left(B-W\right)s_{\theta }\\ \left(W-B\right)c_{\theta }s_{\phi }\\ \left(W-B\right)c_{\theta }c_{\phi } \end{array}\\ \begin{array}{c} z_{B}Bc_{\theta }s_{\phi }\\ z_{B}Bs_{\theta }\\ 0 \end{array} \end{array}\right]$$

## 3. Control System Design

**Figure 6:**
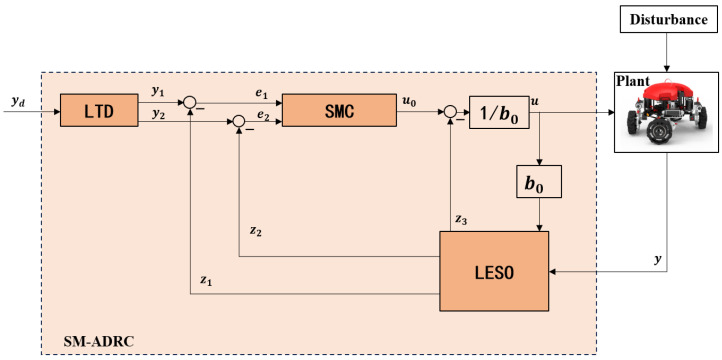
SM-ADRC control block diagram.

This section focuses on the design of the control system. The overall block diagram of the control system is shown in Figure 6. This scheme estimates and compensates for the total disturbance in real time through LESO. Use TD pre-adjust reference instructions with nonlinear piecewise fhan function and suppress overshoot. The SMC integrating LESO disturbance compensation is designed. The integral sliding mode surface and saturation function are adopted to enhance the steady-state accuracy and suppress high-frequency chatters, thereby constructing a control closed loop that is resistant to disturbance and saturation. The detailed design and verification of LESO, TD, and SMC are elaborated in detail in the subsequent sections.

### 3.1. LESO Design

LESO is an important component of linear active disturbance rejection control. It estimates the uncertainties of the internal model of the system, changes in controller parameters, and external disturbances. Regard them as a total disturbance, compensate for the system through feedback, and observe the state variables of the system. The LESO design in this paper refers to Professor Gao’s theory and provides the basis for proof of stability [22].

The second-order nonlinear system of the multi-modal underwater robot can be expressed as Equation (15).

(15) $$\ddot{y}=f\left(y,\dot{y},d\right)+b_{0}u$$

Among them, y is the system output, $u=\tau_{prop}$ is the control input, d is the bit unknown disturbance, f is the total disturbance received by the system, and $b_{0}$ is the compensation coefficient. The state equation is established with any one degree of freedom, as shown in Equation (16).

(16) $$\left\{\begin{array}{c} \dot{x_{1}}=x_{2}\\ \dot{x_{2}}=f+b_{0}u\\ y=x_{1} \end{array}\right.$$

Expand the third state of the original system to $x_{3}=f$. Suppose the differential ℎ of *f* has an upper bound, and let $\dot{f}=h$. Then, the extended state equation of the original system can be expressed as Equation (17).

(17) $$\left\{\begin{array}{c} \dot{x_{1}}=x_{2}\\ \dot{x_{2}}=f+b_{0}u\\ \dot{x_{3}}=h\\ y=x_{1} \end{array}\right.$$

Equation (17) can be further written in the form of Equation (18).

(18) $$\left\{\begin{array}{c} \dot{x}=Ax+Bu+Eh\\ y=Cx \end{array}\right.$$

Among them, $A=\left[\begin{array}{ccc} 0 & 1 & 0\\ 0 & 0 & 1\\ 0 & 0 & 0 \end{array}\right]$, $B=\left[\begin{array}{c} 0\\ b_{0}\\ 0 \end{array}\right]$, $C=\left[\begin{array}{ccc} 1 & 0 & 0 \end{array}\right],$ and $E=\left[\begin{array}{c} 0\\ 0\\ 1 \end{array}\right]$ are coefficient matrices.

The LESO is established through the equation of state, as shown in Equation (19).

(19) $$\left\{\begin{array}{c} e=z_{1}-y\\ \dot{z_{1}}=z_{2}-l_{1}e\\ \dot{z_{2}}=z_{3}-l_{2}e+b_{0}u\\ \dot{z_{3}}=-l_{3}e \end{array}\right.$$

Write Equation (19) further in the form of Equation (20). In the formula, $z={\left[z_{1},z_{2},z_{3}\right]}^{T}$ are the state quantities of LESO, and $L={\left[l_{1},l_{2},l_{3}\right]}^{T}$ are the feedback gain quantities of LESO.

(20) $$\left\{\begin{array}{c} \dot{z}=Az+Bu+L(\hat{y}-y)\\ \hat{y}=Cz \end{array}\right.$$

In order to make parameter tuning more convenient in practical applications, the parameter tuning strategy based on the bandwidth proposed by Professor Gao Zhiqiang is referred to for tuning the observer parameters $l_{1}$, $l_{2}$, and $l_{3}$. The characteristic equation of the observer system is expressed in Equation (21).

(21) $$\lambda \left(s\right)=s^{3}+l_{1}s^{2}+l_{2}s+l_{3}=0$$

When all the observer poles are configured at $-\omega_{0}$, Equation (22) is obtained. Then, the relationship between the bandwidth of the observer and the gain of the observer is obtained as $l_{1}=3\omega_{0}$, $l_{2}=3\omega_{0}^{2}$, and $l_{3}=\omega_{0}^{3}$.

(22) $$s^{3}+l_{1}s^{2}+l_{2}s+l_{3}={\left(s+\omega_{0}\right)}^{3}$$

The configurable parameter of the observer is $\omega_{0}$. The observation effect of the observer on the system state can be adjusted by adjusting the observer bandwidth $\omega_{0}$. The larger the bandwidth, the better the tracking effect on the system state, but the more sensitive it is to disturbances. Therefore, it is necessary to reasonably adjust $\omega_{0}$ to keep the dynamic response and anti-disturbance capability of the system within a relatively acceptable range.

### 3.2. TD Design

TD is an important part of ADRC, used to track the input signal and extract the differential signal. By adjusting the sampling period and tracking speed factor according to the corresponding system, the impact of input signal jumps on the system can be reduced, and the control quality and system robustness can be improved. The tracking differentiator can be expressed as Equation (23).

(23) $$\left\{\begin{array}{c} fh=fhan(x_{1}\left(k\right)-x_{0}\left(k\right),x_{2},r,h)\\ x_{1}\left(k+1\right)=x_{1}\left(k\right)+{hx}_{2}\left(k\right)\\ x_{2}\left(k+1\right)=x_{2}\left(k\right)+hfh \end{array}\right.$$

In the formula, $x_{1}\left(k\right)$ is the tracking expected trajectory signal, $x_{2}\left(k\right)$ is the approximate differential signal of the expected trajectory signal, and r is the tracking speed factor. The larger the speed factor, the faster the tracking speed. In practical applications, the reasonable selection of r is related to the system performance. h is the sampling period of the system. The fhan function is shown in Equation (24).

(24) $$\left\{\begin{array}{c} d=rh^{2},a_{0}=hx_{2}\\ y_{a}=x_{1}+a_{0}\\ a_{1}=\sqrt{d(d+8|y_{a}|)}\\ a_{2}=a_{0}+sign(y_{a})(a_{1}-d)/2\\ s_{y}=\left[sign\left(y_{a}+d\right)-sign\left(a-d\right)\right]/2\\ a=\left(a_{0}+y_{a}-a_{2}\right)s_{y}+a_{2}\\ s_{a}=\left[sign\left(a+d\right)-sign\left(a-d\right)\right]/2\\ fhan=-r\left[a/d-sign\left(a\right)\right]s_{a}-rsign(a) \end{array}\right.$$

The traditional fhan function achieves fast tracking through nonlinear feedback. However, its strong nonlinearity outside the boundary layer leads to two problems: when the tracking error approaches zero, the nonlinear term $-r\left[a/d-sign\left(a\right)\right]s_{a}$ introduces high-frequency buffering, causing overshoot, and the differential channel is sensitive to the input noise, which easily leads to noise amplification. Therefore, the improved piecewise fhan function is introduced, as shown in Equation (25).

(25) $$fhan=\left\{\begin{array}{cc} -r\left[\frac{a}{d}-sign\left(a\right)\right]s_{a}-rsign\left(a\right), & |y_{a}|>d\;\\ -\frac{r}{d}y_{a}, & |y_{a}|\leq d \end{array}\right.$$

While retaining the fast nonlinear tracking characteristics of the outer layer of the boundary, where $|y_{a}|>d$, linear damping is introduced within the boundary layer, where $|y_{a}|\leq d,$ to suppress overshoot, while ensuring the continuity and smooth transition of the function at the boundary.

### 3.3. SMC Design

The system tracking error is defined as $e_{1}=y_{1}-z_{1}$, and $e_{2}=y_{2}-z_{2}$. The first-order integral sliding mode surface is selected, as shown in Equation (26). The derivative of s yields the result of Equation (27).

(26) $$s=\lambda e_{1}+\dot{e_{1}}\;(\lambda >0)$$

(27) $$\dot{s}=\lambda \dot{e_{1}}+\ddot{e_{1}}=\lambda \left(\dot{y_{1}}-\dot{z_{1}}\right)+\left(\ddot{y_{1}}-\ddot{z_{1}}\right)$$

$\ddot{z_{1}}=\ddot{y}$ can be obtained by using the condition $z_{1}-y=0$ for the convergence of the observer. Substituting $\ddot{y}=f+b_{0}u$ gives Equation (28).

(28) $$\dot{s}=\lambda \dot{e_{1}}+\ddot{y_{1}}-f-b_{0}u$$

Let $\dot{s}=0$ solve for the expression of the equivalent control term:

(29) $$u_{eq}=\frac{1}{b_{0}}[\lambda \dot{e_{1}}+\ddot{y_{1}}-f]$$

LESO estimates the total disturbance $z_{3}\approx f$, and substituting it into Equation (29) yields the following result:

(30) $$u_{eq}=\frac{1}{b_{0}}[\lambda \dot{e_{1}}+\ddot{y_{1}}-z_{3}]$$

The expression of the exponential approach law is as follows:

(31) $$\dot{s}=-\epsilon sgn\left(s\right)-ks$$

where ϵ represents the switching gain, and k represents the approaching rate. Substituting the approach law into $\dot{s}$ yields Equation (32).

(32) $$-\epsilon sgn\left(s\right)-ks=\lambda \left(\dot{y_{1}}-\dot{z_{1}}\right)+\ddot{y_{1}}-z_{3}-b_{0}(u_{eq}+u_{sw})$$

The simplified expression of the switching control item is as follows:

(33) $$u_{sw}=\frac{1}{b_{0}}[\epsilon sat\left(\frac{s}{\emptyset }\right)+ks]$$

To reduce chattering, $sat\left(\frac{s}{\emptyset }\right)$ is used instead of the signed function, as shown in Equation (34).

(34) $$sat\left(\frac{s}{\emptyset }\right)=\left\{\begin{array}{cc} sgn\left(s\right) & \left|s\right|\geq \emptyset \\ \frac{s}{\emptyset } & \left|s\right|<\emptyset \end{array}\right.$$

where ∅ represents the boundary layer thickness used to smooth the control signal. The expression of the total control input is shown in Equation (35).

(35) $$u=\frac{1}{b_{0}}[\lambda \dot{e_{1}}+\ddot{y_{1}}-z_{3}+\epsilon sat\left(\frac{s}{\emptyset }\right)+ks]$$

Stability proof:

We represent the LESO estimation error as Equation (36).

(36) $$e_{f}=z_{3}-f$$

The estimation error is bounded. There exists a known positive number δ such that:

(37) $$|e_{f}|\leq \delta$$

The magnitude of δ is related to the bandwidth $\omega_{0}$ of LESO. A higher $\omega_{0}$ usually leads to a smaller δ, but it also increases the sensitivity to measurement noise.

Select the Lyapunov function as $V=\frac{1}{2}s^{2}$. After differentiating it, the following expression is obtained:

(38) $$\dot{V}=s\dot{s}=se_{f}-\epsilon s\cdot sat\left(\frac{s}{\phi }\right)-ks^{2}$$

Then, for the outer layer of the boundary,

(39) $$\dot{V}=se_{f}-\epsilon |s|-ks^{2}\leq -|s|(\epsilon -\delta )-ks^{2}$$

ϵ>δ is a sufficient condition to ensure that $\dot{V}$ ≤ 0. The switching gain ϵ must be greater than the bound δ of the estimation error to ensure stability outside the boundary layer.

Then, for the inner layer of the boundary,

(40) $$\dot{V}=se_{f}-\epsilon \frac{s^{2}}{\phi }-ks^{2}$$

For any γ>0, according to Young’s inequality, we obtain

(41) $$se_{f}\leq \frac{s^{2}}{2\gamma }+\frac{\gamma }{2}e_{f}^{2}$$

Using $e_{f}^{2}\leq \delta^{2}$, we can obtain

(42) $$\dot{V}\leq \left(\frac{1}{2\gamma }-\frac{\epsilon }{\phi }-k\right)s^{2}+\frac{\gamma }{2}\delta^{2}$$

If a constant $\alpha =\frac{\epsilon }{\phi }+k-\frac{1}{2\gamma }>0$ is defined, then Formula (42) can be written as

(43) $$\dot{V}\leq -\alpha s^{2}+\frac{\gamma }{2}\delta^{2}$$

When $\alpha s^{2}>\frac{\gamma }{2}\delta^{2}$, $\dot{V}<0$. Solving this condition gives

(44) $$\left|s\right|>\delta \sqrt{\frac{\gamma }{2\alpha }}$$

Ultimately, the system trajectory will converge into a boundary layer, whose size is determined by $\delta \sqrt{\frac{\gamma }{2\alpha }}$. This conclusion strictly guarantees theoretically that the closed-loop system is uniformly ultimately bounded (UUB) stable.

## 4. Simulation and Experimental Verification

**Figure 7:**
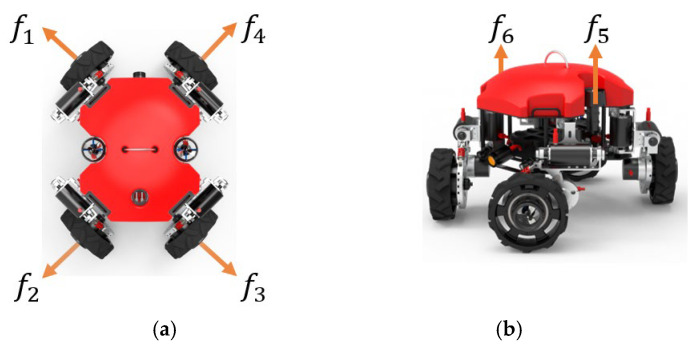
Layout of the propeller. (**a**) The distribution of horizontal thrusters; (**b**) the distribution of vertical thrusters.

The thruster system of the multi-modal underwater robot consists of six underwater thrusters with a maximum thrust of 137 N. The layout of the thrusters is shown in Figure 7.

The thrust matrix is determined based on the positions of each thruster in the robot’s body coordinate system relative to the origin. The thrust vector f is mapped to the generalized force and moment vectors through matrix B, as shown in Equation (45).

(45) τ=Bf

According to the layout of the thruster system in different modes and the positional relationship of each thruster in the robot relative to the origin of the body coordinate system, the specific form of the thrust matrix B in the vector propulsion mode of Equation (45) is shown in Equation (46). In quadrotor mode, as shown in Equation (47). The specific expression of thrust f is shown in Equation (48).

(46) $$B=\left[\begin{array}{cc} \begin{array}{ccc} \;0.7071\; & -0.7071\; & -0.7071\\ \;0.7071\; & 0.7071\; & -0.7071\\ 0 & 0\; & 0 \end{array} & \begin{array}{ccc} \;0.7071\; & 0 & \;0\\ -0.7071\; & 0 & \;0\\ 0\; & 1 & \;1 \end{array}\\ \begin{array}{ccc} 0.117\; & \;0.117 & -0.117\\ -0.117 & \;0.117 & \;0.117\\ 0.122\; & -0.122 & \;0.122 \end{array} & \begin{array}{ccc} -0.117 & \;0.242 & 0.242\\ -0.117 & \;0 & 0\\ -0.122 & \;0 & 0 \end{array} \end{array}\right]$$

(47) $$B=\left[\begin{array}{cc} \begin{array}{ccc} \;0\; & \;0\; & \;0\;\\ \;0\; & \;0\; & \;0\;\\ \;1\; & \;1\; & \;1\; \end{array} & \begin{array}{ccc} \;0\; & \;0\; & \;0\\ \;0\; & \;0\; & \;0\\ \;1\; & \;1\; & \;1 \end{array}\\ \begin{array}{ccc} 0.166 & \;0.166\; & -0.166\;\\ -0.166\; & \;0.166\; & \;0.166\;\\ \;0\; & \;0\; & \;0\; \end{array} & \begin{array}{ccc} -0.166 & \;0.242 & -0.242\\ -0.166 & \;0 & \;0\\ 0\; & \;0 & \;0 \end{array} \end{array}\right]$$

(48) $$f=\left[f_{1},f_{2},f_{3},f_{4},f_{5},f_{6}\right]$$

This section builds a multi-modal underwater robot motion control system based on the MATLAB R2023b platform. Based on the mathematical model established in Chapter 2, the heading control and depth control simulation of the vector propulsion mode of the multi-modal underwater robot and the depth control simulation of the quadrotor mode are carried out. Given the high consistency between the verification results of the basic functions of the still water environment and the simulation experiment results, this study adopts a simulation experiment to verify the universality of the algorithm. The verification of the offshore prototype will be carried out after the algorithm has completed sufficient simulation tests, the experimental plan has been formulated in detail, and a sound risk prevention and control mechanism has been established, in order to minimize the risk of loss in the early physical tests to the greatest extent.

To verify the anti-disturbance capability of the algorithm, this paper introduces time-varying external disturbances that are independent of the controller state during the robot control process, aiming to evaluate the dynamic response of the control system. Meanwhile, time-varying disturbances related to the robot’s state are introduced, aiming to verify the robustness of the robot in dynamic marine environments and the effectiveness of chattering suppression.

### 4.1. Vector Propulsion Modal Heading and Depth Control

#### 4.1.1. Heading and Depth Control Without Disturbance

**Figure 8:**
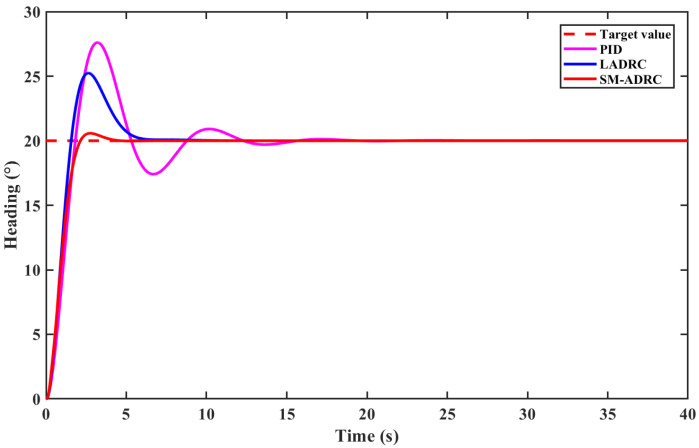
Simulation results of vector propulsion modal heading control without disturbance.

Figure 8 shows the directional control of the multi-modal underwater robot with a heading angle of 20° and compares the control effects of SM-ADRC, LADRC, and PID. The setting parameters of the three control algorithms are shown in Table 2 and Table 3.

When adjusting the SM-ADRC parameters, the estimated value of $b_{0}$ is first obtained by using the system model estimation, and then $b_{0}$ is gradually adjusted. After converging the control system, gradually adjust $\omega_{0}$ to make the control system tend to be stable and free of oscillation. λ is the parameter of the sliding mode surface, which is preliminarily determined based on the expected error convergence time. Under the premise of ensuring the stability of the system, gradually increase λ to improve the response speed. When adjusting k, first set a smaller value and gradually increase it according to the response speed. Adjust k to obtain a satisfactory approach velocity, and then adjust ϵ to suppress the disturbance. The boundary layer thickness ∅ is set to a larger initial value first. Then, gradually decrease ∅. The robustness and accuracy of the system will improve, but high-frequency components will begin to appear in the control signal. When the high-frequency chattering of the control output reaches the allowable limit of the thruster, stop decreasing ∅.

Analyze the dynamic performance of directional control without external disturbance, based on Figure 8 and Table 4. The piecewise fhan function attributed to TD effectively suppresses overshoot, and the overshoot of SM-ADRC is within 3%, which is a significant improvement compared with traditional PID and LADRC controllers. The regulation time of SM-ADRC was 3.9 s, which was shortened by 34.0% and 73.0%, respectively, compared with 5.91 s of LADRC and 14.44 s of PID. In terms of steady-state performance, the fluctuation amplitude of SM-ADRC is significantly smaller than that of PID and LADRC, effectively suppressing chattering. It can be seen that SM-ADRC has an excellent dynamic response for the directional control of multi-modal underwater robots, verifying the effectiveness of the SM-ADRC control algorithm for directional control.

Figure 7 shows the depth control effect of the multi-modal underwater robot diving from the water surface to a depth of 10 m under undisturbed conditions, and compares SM-ADRC, LADRC, and traditional PID. The setting parameters of the control algorithm are shown in Table 5 and Table 6.

**Figure 9:**
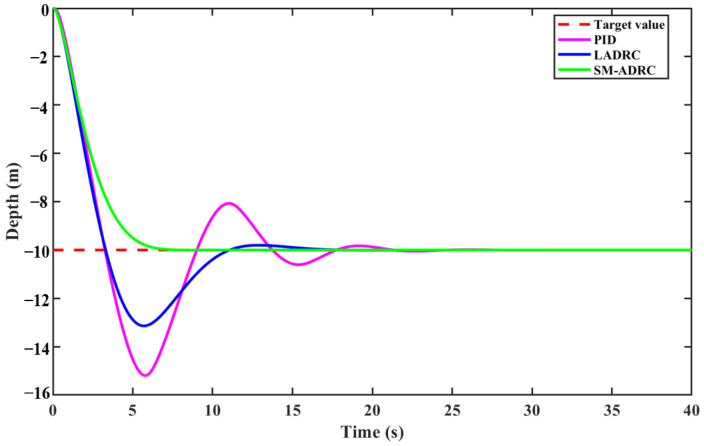
Simulation results of vector propulsion modal depth control without disturbance.

According to the analysis in Figure 9 and Table 7, SM-ADRC achieves zero overshoot, which has a significant advantage compared with the 31.41% overshoot of LADRC and the 51.95% overshoot of traditional PID. The regulation time of SM-ADRC is the shortest, which is 43.9% shorter than that of LADRC at 10.31 s and 66.1% shorter than that of PID at 17.06 s. In terms of steady-state performance, compared with PID and LADRC, SM-ADRC has a smaller fluctuation amplitude and a significant suppression effect on chattering. The comprehensive results show that SM-ADRC has a better dynamic response for the depth control of multi-modal underwater robots.

#### 4.1.2. Heading and Depth Control in Case of Disturbance

**Figure 10:**
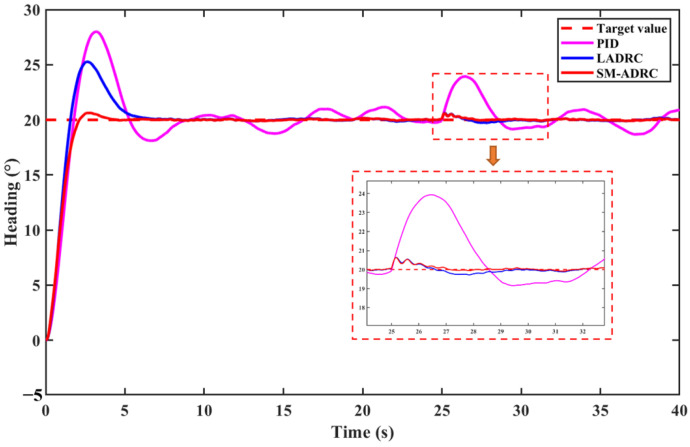
Anti-disturbance simulation results of vector propulsion modal heading control.

**Figure 11:**
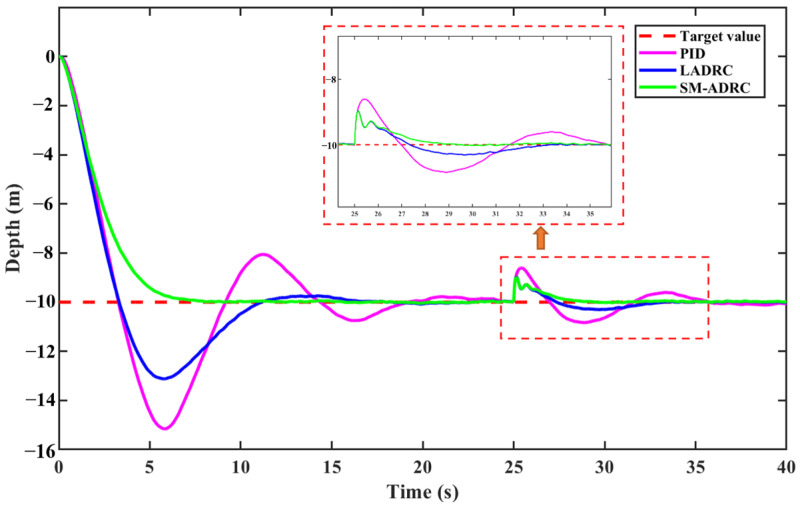
Anti-disturbance simulation results of vector propulsion modal depth control.

Based on Section 4.1.1, a random white noise perturbation with a bandwidth of 5 Hz and an amplitude of ±10 N is added to simulate the continuous random external perturbations that the robot experiences during the control process. At 25 s, a disturbance force of 200 N is applied to simulate the robot being suddenly disturbed by a large external force. While keeping the other control parameters unchanged, the dynamic response in the presence of disturbance is obtained, as shown in Figure 10 and Figure 11.

It can be seen from Figure 8 that all three controllers are affected by random white noise. PID has the largest fluctuation, LADRC has a smaller fluctuation, and SM-ADRC has the smallest fluctuation. After applying a perturbation step, the perturbation capabilities of the different controllers also vary. SM-ADRC shows significant robustness when subjected to a 200 N disturbance force. Its bow angle offset returns to a steady state within 3 s, and the recovery speed is greatly improved compared with LADRC and PID. Meanwhile, the maximum offset angle caused by disturbance is the smallest. The control effect of the PID controller shows a significant deviation, indicating that the anti-disturbance ability of LADRC is superior to that of PID, while the anti-disturbance ability of SM-ADRC is improved on the basis of LADRC. Taking all the above factors into consideration, SM-ADRC has the best control effect and the most optimal performance.

It can be seen from the simulation results in Figure 9 that the fluctuation of PID control is the most significant, showing a large oscillation. LADRC shows a significant improvement in depth control accuracy compared to PID, and the fluctuation amplitude is significantly reduced. While SM-ADRC demonstrates excellent disturbance suppression capability, its depth curve is the smoothest and most stable. When the system is subjected to a disturbance force of 200 N, SM-ADRC demonstrates outstanding robustness, with the smallest depth deviation amplitude and the fastest recovery to the target value among the three controllers. Although the LADRC controller has better anti-disturbance characteristics than PID, it is inferior to SM-ADRC in terms of transient recovery performance and overshoot suppression. The comprehensive results show that SM-ADRC exhibits the best performance in terms of anti-disturbance ability and dynamic response in the depth-fixing control task.

### 4.2. Quadrotor Modal Depth Control

For the fully vertical thruster configuration unique to the quadrotor modal, this section focuses on the single-channel control characteristics of vertical degrees of freedom. Through depth control simulation, the dynamic performance of SM-ADRC, LADRC, and PID under undisturbed conditions is compared, and the response rapidity and control accuracy of the algorithm in vertical plane motion are systematically verified.

#### 4.2.1. Depth Control of the Quadrotor Modal Without Disturbance

**Figure 12:**
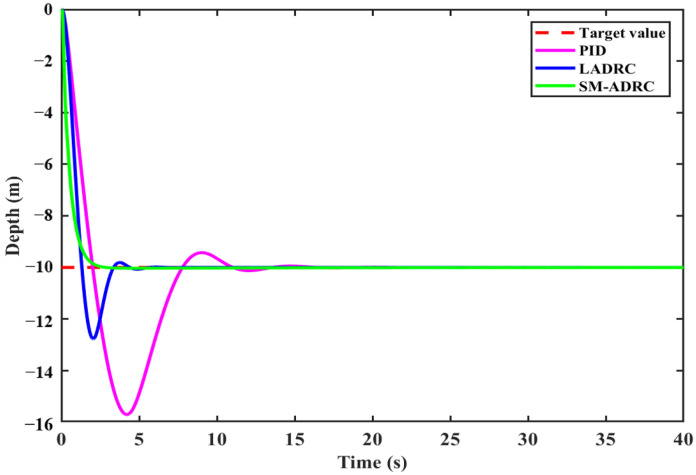
Simulation results of quadrotor modal depth control without disturbance.

Figure 12 shows the dynamic response comparison curves of the 10 m constant depth simulation of three controllers in the quadrotor mode under undisturbed conditions. The setting parameters of the three control algorithms are shown in Table 8 and Table 9.

SM-ADRC demonstrates significant advantages in the depth control of quadrotor modal, and the quantitative indicators are shown in Table 10. By analyzing the data in the table, it can be seen that SM-ADRC achieves monotonic convergence without overshoot and has significant advantages compared with PID and LADRC. The regulation time is 2.49 s, which is 53.1% lower than 5.31 s of LADRC and 82.0% lower than 13.83 s of PID. Moreover, the overall response speed of the quadrotor modal is faster than that of the vector propulsion mode. As can be seen from the above, SM-ADRC has excellent performance in the depth control of robots in the quadrotor modal.

#### 4.2.2. Quadrotor Modal Depth Tracking

**Figure 13:**
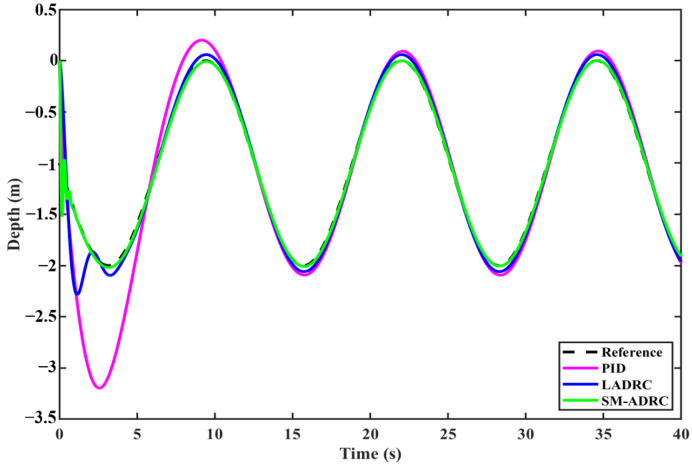
Simulation results of quadrotor modal depth tracking.

**Figure 14:**
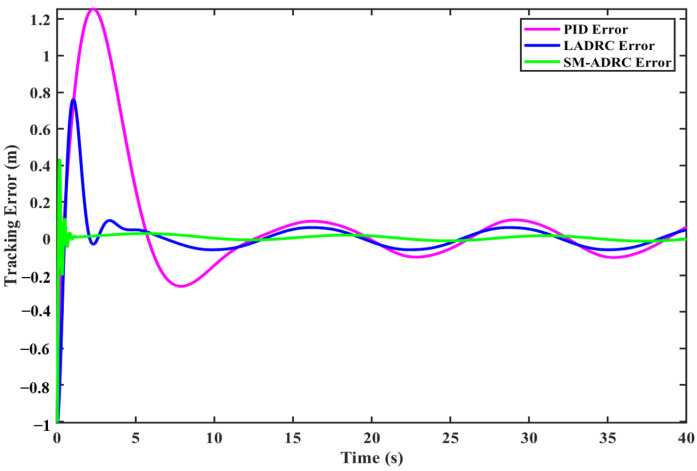
Quadrotor modal depth tracking error.

To comprehensively evaluate the depth tracking performance of the controller, the reference depth was set to the time-varying sine function Ref = 1 + sin(*t*), and the depth tracking experiment was tuned based on the parameters in Section 4.2.1. Figure 13 shows the depth tracking curves of different controllers. Analysis shows that the fluctuation range of SM-ADRC is significantly smaller than that of LADRC and PID, and its convergence speed is the fastest. Figure 14 shows the tracking error of the controller. When the system is stable, the error of SM-ADRC is the smallest, and the error of LADRC is similar to that of PID, but relatively larger. The experiment intuitively proved that SM-ADRC has obvious advantages in reducing fluctuations, accelerating convergence, and minimizing steady-state errors in deep tracking tasks.

Table 11 presents a detailed comparison of error data. The average tracking deviation of SM-ADRC is 0.0046 m, which is 52.6% lower than that of LADRC at 0.0097 m and 93.7% lower than that of PID at 0.0729 m. The root mean square error of SM-ADRC was 0.0471 m, which was increased by 64.7% and 85.6%, respectively, compared with 0.1333 m of LADRC and 0.3268 m of PID. The comparison and verification of these two indicators show that SM-ADRC achieves higher steady-state tracking accuracy.

#### 4.2.3. Depth Control of the Quadrotor Modal in Case of Disturbance

**Figure 15:**
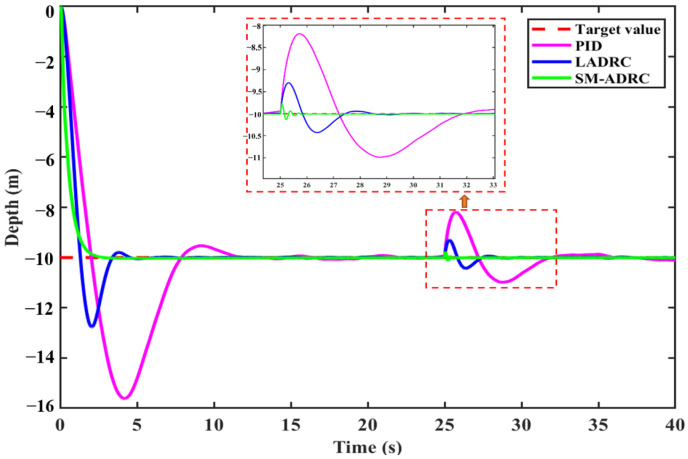
Anti-disturbance simulation results of quadrotor modal depth control.

To quantitatively evaluate the anti-disturbance capability of SM-ADRC in the quadrotor mode, a compound disturbance consistent with that in Section 4.1.2 was applied to the system. The depth response curve of the system under disturbance is shown in Figure 15.

The simulation results show that SM-ADRC has the smallest depth fluctuation range, and its anti-disturbance ability is superior to that of LADRC and PID. When subjected to a large disturbance force of 200 N, SM-ADRC demonstrates stronger robustness. Its recovery speed is faster, adjusting to a steady state within 1.0 s. The recovery speed is 75.0% and 85.7% higher than that of LADRC at 4.0 s and PID at 7.0 s, respectively. Overall, SM-ADRC demonstrates the best comprehensive anti-disturbance performance and robustness.

## 5. Conclusions

This paper proposes an SM-ADRC scheme in view of problems such as model uncertainty and external disturbances existing in the operation process of multi-modal underwater robots. Firstly, LESO is constructed to estimate and compensate for model uncertainties and external disturbances in real time. Secondly, a TD based on a nonlinear piecewise fhan function is designed to pre-adjust the given reference instruction, effectively suppressing the tracking overshoot caused by sudden instruction changes. Finally, the SMC is designed, whose control law integrates the total disturbance estimate provided by LESO for feedforward compensation. Meanwhile, the integral sliding mode surface is adopted to enhance steady-state accuracy, and the continuous saturation function is introduced to replace the symbolic function, significantly weakening the control input chattering, thereby forming a complete SM-ADRC anti-disturbance and anti-saturation control framework.

In this paper, a simulation model of a multi-modal underwater robot in an environment with multiple disturbances is constructed in the MATLAB R2023b environment. For the tasks of heading control and depth control, the performance of the SM-ADRC proposed in this paper, LADRC and PID in key performance indicators such as control accuracy, anti-disturbance ability, and robustness was systematically compared and analyzed. The simulation results show that in both vector propulsion and quadrotor modes, the proposed SM-ADRC scheme demonstrates significant advantages over LADRC and PID in terms of dynamic tracking accuracy, disturbance suppression ability, and robustness. The core objective of this paper is to provide an immediately available and easy-to-implement engineering solution for the multi-modal underwater robot, which is a highly nonlinear and coupled controlled object. At this point, the scheme demonstrates excellent control performance. However, this scheme will sacrifice some of the robustness of the ideal sliding mode while eliminating chattering. In future work, we will be committed to researching more advanced control strategies that can not only maintain strong robustness but also effectively suppress chattering. We will consider using learning-based parameter tuning methods to adapt to LESO, TD, and SMC, exploit multi-robot cooperative control strategies, and consider multi-sensor fusion. We will promptly advance real-world verification and accelerate the implementation of engineering applications.

## Figures and Tables

**Table 1:** Definition of motion in a coordinate system.

	Position and Angles Earth-Fixed Frame	Linear Velocity and Angular Velocity Body-Fixed Frame	Force and Moments Body-Fixed Frame
Surge	x	u	X
Sway	y	v	Y
Heave	z	w	Z
Roll	ϕ	p	K
Pitch	θ	q	M
Yaw	ψ	r	N

**Table 2:** SM-ADRC heading control parameter setting table.

The Parameter of SM-ADRC	Numerical Value	The Parameter of SM-ADRC	Numerical Value
λ	3	*k*	1.1
ϵ	1.5	$\omega_{0}$	12
∅	0.6	$b_{0}$	0.046

**Table 3:** LADRC and PID heading control parameter setting table.

The Parameter of LADRC	Numerical Value	The Parameter of PID	Numerical Value
$\omega_{0}$	8	$k_{p}$	18
$\omega_{c}$	3	$k_{i}$	4
$b_{0}$	0.046	$k_{d}$	10

**Table 4:** Dynamic response performance indicators of vector propulsion modal heading control.

Indicators	SM-ADRC	LADRC	PID
$t_{r}$	2.12 s	1.56 s	1.82 s
$t_{s}\;\;(∆=0.02)$	3.90 s	5.91 s	14.44 s
σ%	2.81%	26.24%	38.05%
$e_{ss}$	0.01°	0.01°	0.01°

**Table 5:** SM-ADRC depth control parameter setting table.

The Parameter of SM-ADRC	Numerical Value	The Parameter of SM-ADRC	Numerical Value
λ	3	*k*	2.6
ϵ	0.3	$\omega_{0}$	8
∅	0.4	$b_{0}$	0.046

**Table 6:** LADRC and PID depth control parameter setting table.

The Parameter of LADRC	Numerical Value	The Parameter of PID	Numerical Value
$\omega_{0}$	8	$k_{p}$	25
$\omega_{c}$	2.5	$k_{i}$	6.8
$b_{0}$	0.046	$k_{d}$	10

**Table 7:** Dynamic response performance indicators of vector propulsion modal depth control.

Indicators	SM-ADRC	LADRC	PID
$t_{r}$	5.85 s	3.31 s	3.25 s
$t_{s}\;\;(∆=0.02)$	5.78 s	10.31 s	17.06 s
σ%	0%	31.41%	51.95%
$e_{ss}$	0.01°	0.01°	0.01°

**Table 8:** SM-ADRC depth control parameter setting table.

The Parameter of SM-ADRC	Numerical Value	The Parameter of SM-ADRC	Numerical Value
λ	3	*k*	3.5
ϵ	2.6	$\omega_{0}$	9
∅	0.8	$b_{0}$	0.033

**Table 9:** LADRC and PID depth control parameter setting table.

The Parameter of LADRC	Numerical Value	The Parameter of PID	Numerical Value
$\omega_{0}$	8	$k_{p}$	18
$\omega_{c}$	6.5	$k_{i}$	9
$b_{0}$	0.033	$k_{d}$	12

**Table 10:** Dynamic response performance indicators of quadrotor modal depth control.

Indicators	SM-ADRC	LADRC	PID
$t_{r}$	5.85 s	3.31 s	3.25 s
$t_{s}\;\;(∆=0.02)$	5.78 s	10.31 s	17.06 s
σ%	0%	31.41%	51.95%
$e_{ss}$	0.01°	0.01°	0.01°

**Table 11:** Comparison of deep tracking performance indicators.

Controller	Avg(m)	RMSE(m)
SM-ADRC	0.0046	0.0471
LADRC	0.0097	0.1333
PID	0.0792	0.3268

## Data Availability

The data presented in this study are available on request from the corresponding author.
